# G0 arrest gene patterns to predict the prognosis and drug sensitivity of patients with lung adenocarcinoma

**DOI:** 10.1371/journal.pone.0309076

**Published:** 2024-08-19

**Authors:** Yong Ma, Zhilong Li, Dongbing Li, Baozhen Zheng, Yanfeng Xue

**Affiliations:** 1 Thoracic Surgery Department, Shanxi Province Cancer Hospital/Shanxi Hospital Affiliated to Cancer Hospital, Chinese Academy of Medical Sciences/Cancer Hospital Affiliated to Shanxi Medical University, Taiyuan City, Shanxi, China; 2 Scientific Research Center, Beijing ChosenMed Clinical Laboratory Co., Ltd., Beijing, China; 3 Radiation Oncology Department, Shanxi Province Cancer Hospital/Shanxi Hospital Affiliated to Cancer Hospital, Chinese Academy of Medical Sciences / Cancer Hospital Affiliated to Shanxi Medical University, Taiyuan, Shanxi, China; 4 Special Need Medical Department, Shanxi Province Cancer Hospital/Shanxi Hospital Affiliated to Cancer Hospital, Chinese Academy of Medical Sciences/Cancer Hospital Affiliated to Shanxi Medical University, Taiyuan, Shanxi, China; The University of Texas, MD Anderson Cancer Center, UNITED STATES OF AMERICA

## Abstract

G0 arrest (G0A) is widely recognized as a crucial factor contributing to tumor relapse. The role of genes related to G0A in lung adenocarcinoma (LUAD) was unclear. This study aimed to develop a gene signature based on for LUAD patients and investigate its relationship with prognosis, tumor immune microenvironment, and therapeutic response in LUAD. We use the TCGA-LUAD database as the discovery cohort, focusing specifically on genes associated with the G0A pathway. We used various statistical methods, including Cox and lasso regression, to develop the model. We validated the model using bulk transcriptome and single-cell transcriptome datasets (GSE50081, GSE72094, GSE127465, GSE131907 and EMTAB6149). We used GSEA enrichment and the CIBERSORT algorithm to gain insight into the annotation of the signaling pathway and the characterization of the tumor microenvironment. We evaluated the response to immunotherapy, chemotherapy, and targeted therapy in these patients. The expression of six genes was validated in cell lines by quantitative real-time PCR (qRT-PCR). Our study successfully established a six-gene signature (CHCHD4, DUT, LARP1, PTTG1IP, RBM14, and WBP11) that demonstrated significant predictive power for overall survival in patients with LUAD. It demonstrated independent prognostic value in LUAD. To enhance clinical applicability, we developed a nomogram based on this gene signature, which showed high reliability in predicting patient outcomes. Furthermore, we observed a significant association between G0A-related risk and tumor microenvironment as well as drug susceptibility, highlighting the potential of the gene signature to guide personalized treatment strategies. The expression of six genes were significantly upregulated in the LUAD cell lines. This signature holds the potential to contribute to improved prognostic prediction and new personalized therapies specifically for LUAD patients.

## Introduction

Lung cancer is a highly prevalent malignant tumor worldwide, responsible for approximately one-fourth of all cancer-related deaths in 2021 [1]. Among non-small cell lung cancers, lung adenocarcinoma (LUAD) constitutes more than half of the cases. However, early diagnosis and personalized therapy for LUAD still pose significant challenges [2]. Currently, surgery, radiotherapy, chemotherapy, targeted therapy, and immunotherapy are the main clinical treatments, and progress has been made [3]. Despite the advances in treatment, the prognosis of lung cancer remains bleak, with a 5-year survival rate of less than 10% [4]. Therefore, there is an urgent need to establish more effective prognostic models to improve prediction accuracy and clinical outcomes.

G0 arrest (G0A) is widely recognized as a crucial factor contributing to tumor relapse [5]. Malignant tumor cells in G0A possess the ability to evade immune responses and various mechanisms of cell death [6, 7]. Despite the widespread appearance of G0A in different types of cells, the specific role of individual genes associated with G0A in cancer prognosis and progression remains poorly defined [8, 9]. A challenge in understanding this phenomenon is the heterogeneity of G0A. G0A can be induced by various methods such as serum starvation, mitogen withdrawal, or contact inhibition [10]. Furthermore, G0A can also occur spontaneously in response to intrinsic cellular factors such as replication stress [11–13]. The clinical significance of G0A-related genes in LUAD requires further study. Therefore, more research is needed to fill this gap.

Our primary objective is to comprehensively assess the associations between G0A-related genes and various subtypes of LUAD, mutation profiles, and tumor microenvironment (TME) and validate the expression of six genes was validated in cell lines by quantitative real-time PCR (qRT-PCR). Furthermore, we developed a risk scoring model based on G0A genes, which offers promising prospects for the advancement of personalized and precise therapeutic approaches.

## Materials and methods

### Data collection

The multiomics data from the TCGA-LUAD program was collected, including transcriptome profiles, copy number variations, somatic mutation data and comprehensive clinical characteristics of patients, which were processed using the “TCGAbiolinks” package [14]. Clinical information and gene-level copy number profiles were downloaded from UCSC XENA (https://xenabrowser.net/). Using these comprehensive data sets and analysis tools, our aim is to gain a deeper understanding of the molecular landscape and genetic alterations associated with LUAD, ultimately contributing to advancements in the diagnosis and treatment of this disease [15, 16]. The 500 LUAD samples we obtained from TCGA and the corresponding clinical data. To further investigate, the patients were randomly divided into a training cohort (n = 250) and a test cohort (n = 250) in a 1:1 ratio. We also obtained comprehensive information from two additional cohorts from the Gene Expression Omnibus (GSE50081 and GSE72094).

The data utilized in this study were obtained from the TCGA and GEO databases, which offer unrestricted access to data for research and publication purposes. As a result, ethical approval or patient consent was not required for this study. Notably, this study exclusively utilized cell lines and did not involve the use of human or animal tissues.

### Consensus clustering analysis

The “ConsensusClusterPlus” package was applied to identify distinct unidentified G0A-related patterns of LUAD by k-means algorithms. To identify the optimal number of clusters, the stability of the results was assessed for various cluster numbers ranging from k = 2 to k = 9, with the following specific parameters: repeat 100 times, extract 80% of the total sample, clusterAlg = “hc”, innerLinkage = “ward.D2”.

### Molecular patterns and functional enrichment analysis

Using single sample gene set enrichment analysis (ssGSEA), we identified functional enrichment in Kyoto Encyclopedia of Genes and Genomes (KEGG) related signaling pathways [17].

### Establishment of prognostic model

We assessed the score for each sample in both the training and validation sets using the developed model. The criteria for dividing patients into high-risk and low-risk categories is to divide them into two groups based on their risk scores. To evaluate the prognostic significance of G0A, we use the Kaplan-Meier survival curve and the receiver operating characteristic curve [18, 19].

### Establishment of a nomogram

A prediction nomogram was created to provide patients with LUAD with valuable clinical parameters, risk scores and additional variables, including particularly 1-, 3- and 5-year OS, as well as associated calibration plots [20–23]. The calibration index was applied to assess the ability of all prognostic signatures to predict the prognosis in each cohort (R package “survminer” and “rms”). A concordance index plot (R package ’rms’ and ’survminer’) was generated to compare the cumulative hazard [24]. A Decision Curve Analysis (DCA) was used (R package “survminer” and “ggDCA”) to predict the 1-, 3-, and 5- year OS [25].

### Analysis of TME characterization and prediction of immunotherapy response

We used the CIBERSORT algorithm and the tumor immune dysfunction and exclusion (TIDE) algorithm to estimate the potential for tumor immune infiltrate and immune escape profiles based on gene expression and to predict response to immunotherapy [26, 27]. The stromal, immune and ESTIMATE scores were compared with the “estimate” R package [28]. Unpaired t-test analysis. The algorithm was applied to analyze the differences between the high- and low-risk groups.

### Drug sensitivity analysis

The difference in drug sensitivity between patients with high and low G0A was analyzed using the "oncoPredict" package in R. Half-maximal inhibitory concentrations (IC50) of the drugs were used to present drug sensitivity data [29].

### Validation in a single-cell dataset

The Tumor Immune Single Cell Hub (TISCH1, http://tisch1.comp-genomics.org/) is a scRNA-seq database that allows exploration of the tumor microenvironment [30]. In this work, we use the GSE146771 dataset to decipher the heterogeneity of TME in various cluster LUAD tumor sites at annotated cluster levels.

### qRT-PCR

Human normal lung epithelial cell (BEAS-2B), lung cancer cell lines (A549 and HCC827), were originally purchased from the cell bank of the Chinese Academy of Sciences. BEAS-2B, A549 and HCC827 cells were cultured in high-glucose DMEM medium (SH30022.01B; HyClone, Beijing, China) supplemented with 10% fetal bovine serum (11011–8611; Sijiqing Biotechnology, Hangzhou, China) and 1% penicillin-streptomycin (3810-74-0; Sigma, USA) at 37 °C in the presence of 5% CO_2_. The levels of CHCHD4, DUT, LARP1, PTTG1IP, RBM14, and WBP11 was evaluated using qRT-PCR in BEAS-2B, A549, and HCC827 cell lines. The sequence of primers is as follows:

CHCHD4-F, 5’-ACCCCAACGATCCATACGAG-3’,
CHCHD4-R, 5’-TCTACACAGTCTGACCCCTT-3’;
DUT-F, 5’-CGCGGGCTACGACCTGT-3’,
DUT-R, 5’-CTATGACACCAGCTCCTACATCA-3’;
LARP1-F, 5’-CCCCGTCAGCACTACCAA-3’,
LARP1-R, 5’-GCCTCTTCTTCACTTCAATCCAGT-3’;
PTTG1IP-F, 5’-AAGCCGGACAGGAGTGAG-3’,
PTTG1IP-R, 5’-CAAATCTAGCATACGGGTTTTCTTC-3’;
RBM14-F, 5’-AGATTTTCGTGGGCAATGTGT-3’,
RBM14-R, 5’-GTTCCACGTTGATGCGCTTG-3’;
WBP11-F, 5’-CGTAGGCGAGATGAAGACATGTT-3’,
WBP11-R, 5’-CACAAATTCATCCCCGTCACT-3’;
GAPDHF1, 5’-TGCACCACCAACTGCTTAGC-3’,
GAPDHR1, 5’-GGCATGGACTGTGGTCATGAG-3’.

### Statistical analysis

All statistical computations and graphics were performed using the R programming language (version 4.2.3) using the necessary packages. The significance cut-off value was set at p < 0.05.

## Results

### Landscape of genetic mutations of G0A in LUAD

**Fig 1:**
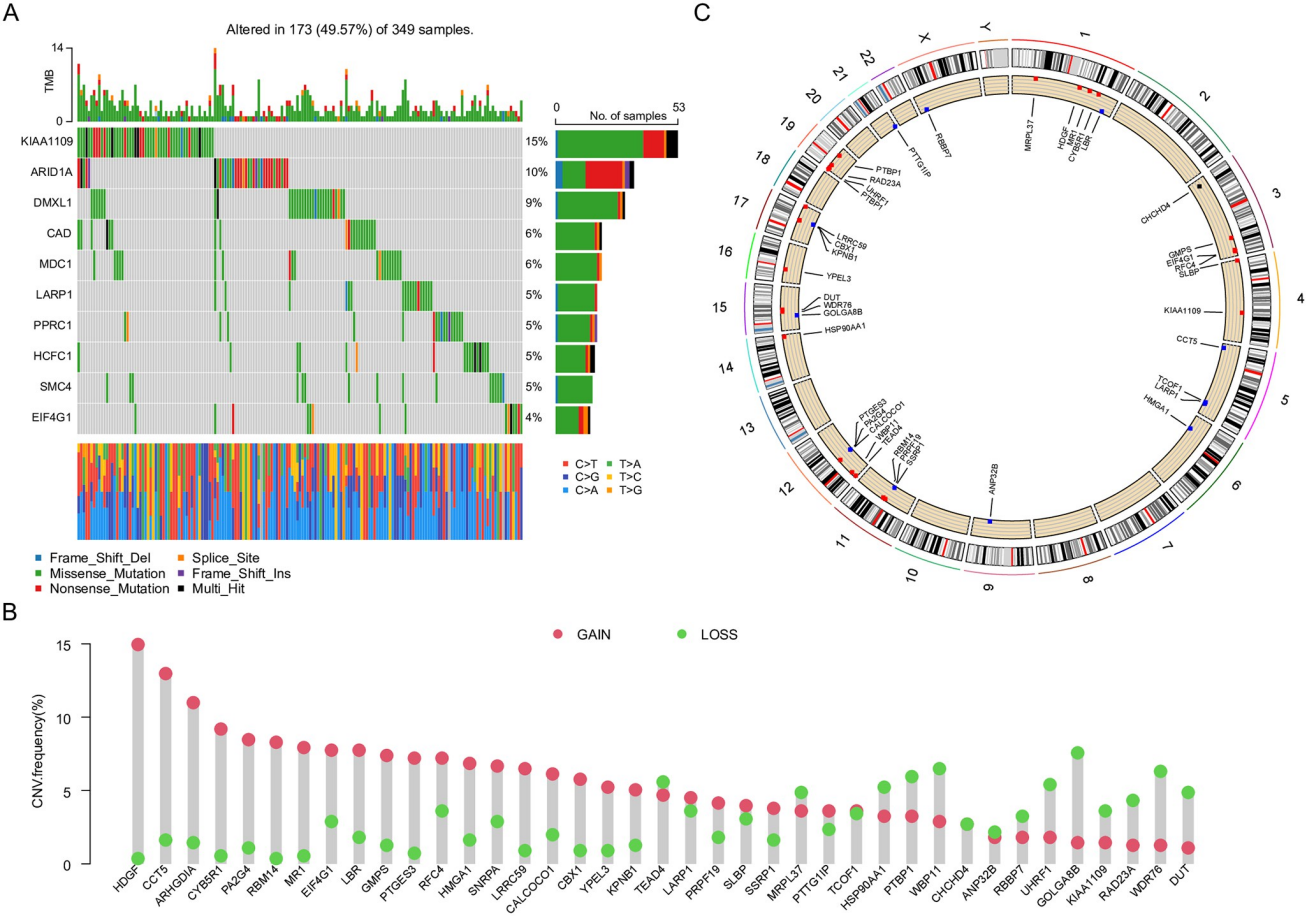
Variant landscape of G0A -related genes in TCGA-LUAD. (A) An analysis of G0A-related genes in the TCGA cohort. (B) Copy number values of G0A-related genes observed in the TCGA cohort. (C) The location and expression of copy number variations in G0A-related genes within the TCGA cohort.

We then performed an uniCox and Kaplan-Meier analysis to assess the prognostic value of these 139 genes related to G0A in patients with LUAD. Our findings revealed that among the 349 LUAD samples analyzed, 173 (or 49.57%) exhibited genetic mutations (Fig 1A). The most mutated gene was KIAA1109, followed by ADID1A, DMXL1, CAD, MDC1, LARP1, PPRC1, HCFC1, SMC4, and EIF4G1. These results highlight significant genomic alterations in LUAD patients and have identified potential driver genes involved in LUAD tumorigenesis. Furthermore, we identified 39 genes with copy number alterations (Fig 1B). The chromosomal locations of these copy number alterations are shown in Fig 1C. These observations suggest that copy number variations may play a regulatory role in G0A-related genes and underscore their potential involvement in the development of LUAD.

### Molecular subtypes related to consensus prognosis of LUAD

**Fig 2:**
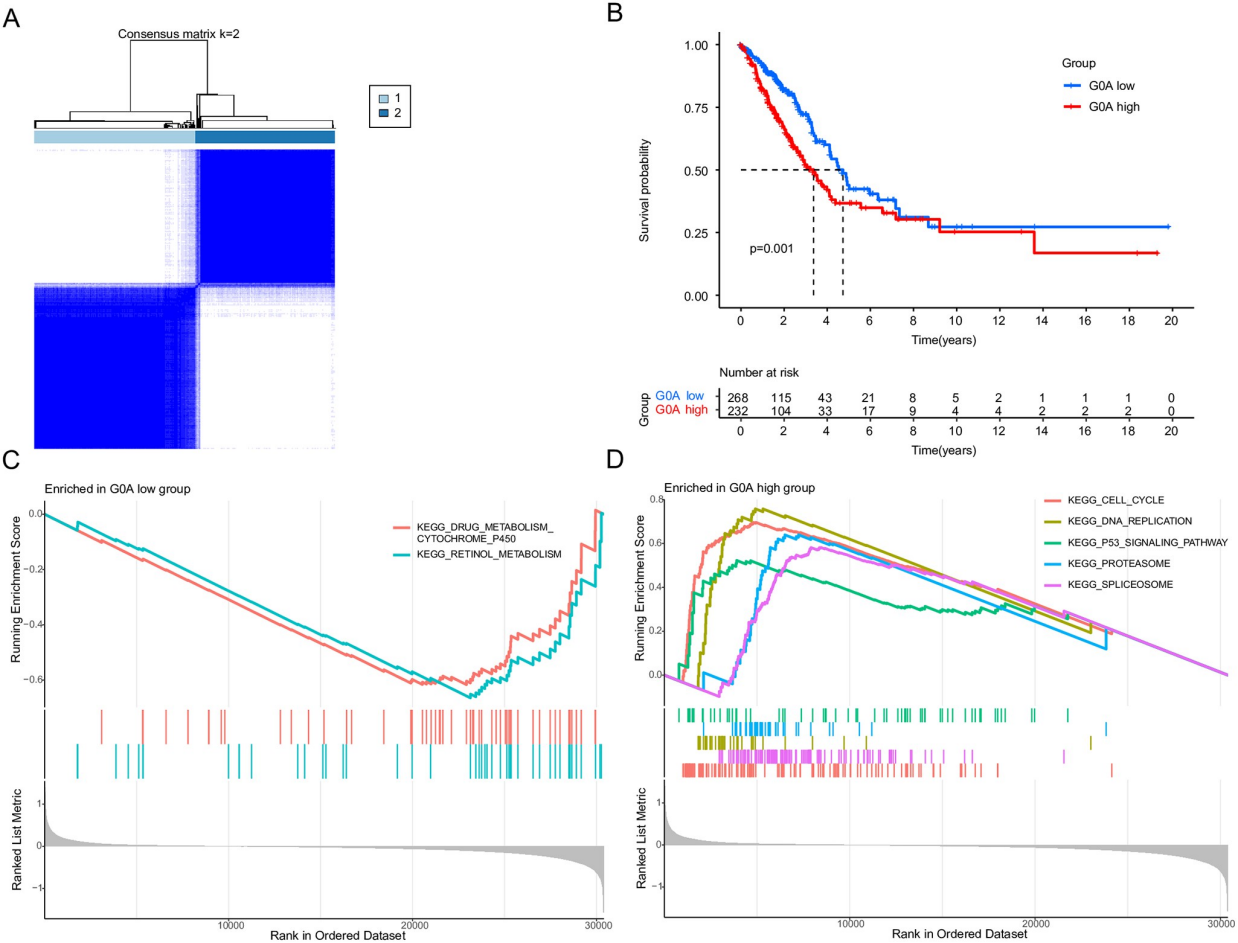
Identification of G0A-associated subtypes by consensus clustering. (A) A heat map illustrating the consensus clustering solution (k = 2) in LUAD samples; (B) Kaplan-Meier curves displaying OS in different G0A-related clusters. (C) GSEA analysis evaluating the enrichment of KEGG in the G0A low group. (D) GSEA analysis that evaluates the enrichment of KEGG in the high G0A group.

Our analysis aimed to classify patients with LUAD into different groups according to their expression levels of G0A. Our analysis identified two as the optimal number of clusters. Consequently, LUAD patients in the combined cohort were classified into group A (n = 268) and group B (n = 232) which demonstrated good dispersion (Fig 2A). Subsequently, principal component analysis results provided further evidence for the observed distinct distributions between clusters, as depicted in Fig 2B. Furthermore, a GSVA analysis revealed that Group A was more enriched in KEGG pathways such as retinol metabolism and cytochrome P450 (Fig 2C); while Group B were related more to these pathways, such as DNA replication, cell cycle, proteasome, spliceosome and P53 signaling pathway (Fig 2D).

### Development and validation of the prognostic model

**Fig 3:**
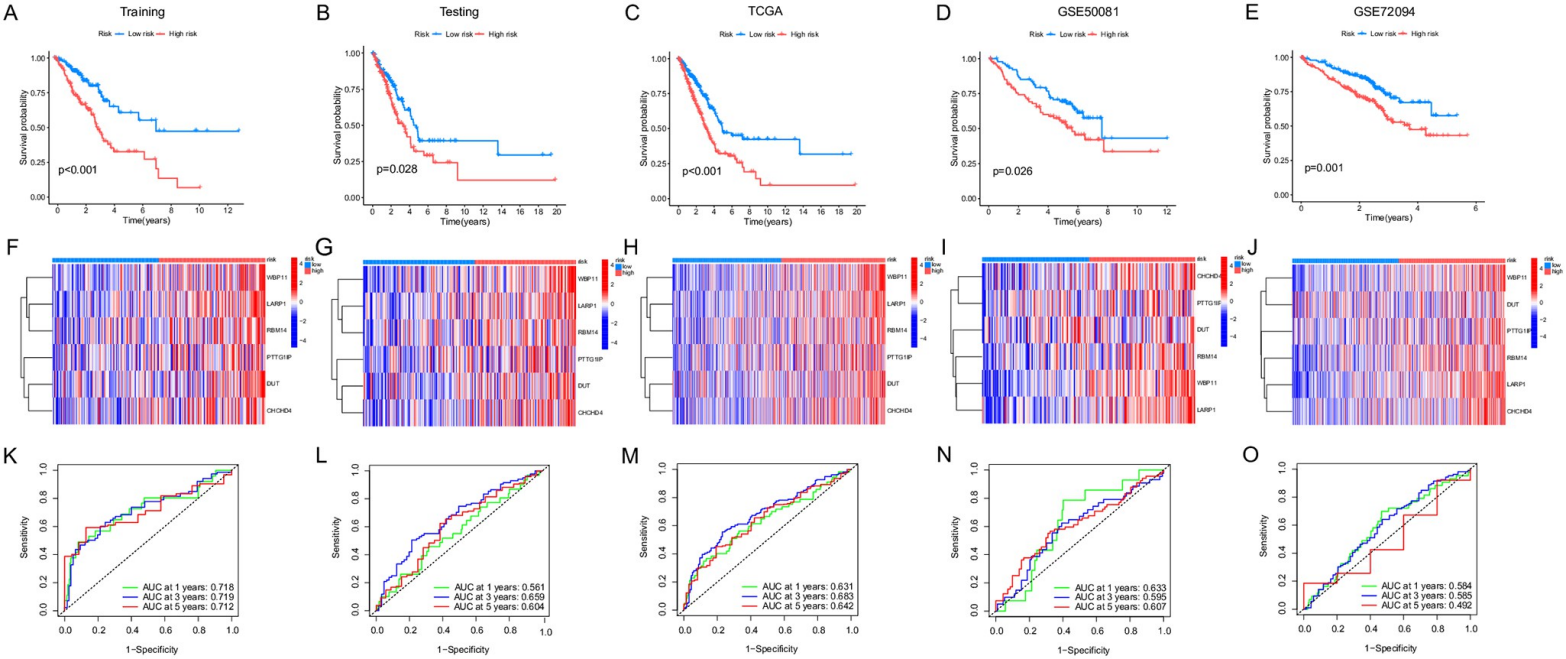
Construction and validation of the prognostic risk model. (A-E) Kaplan-Meier curves displaying OS in the training set, the testing set, the TCGA cohort, the GSE50081 and GSE72094 cohort. (F-J) Heatmaps illustrating the distribution of key signature genes in the training set, test set, TCGA cohort, GSE50081, and GSE72094 cohort. (K-O) ROC curves illustrating the performance of the risk score in the training set, testing set, TCGA cohort, GSE50081 and GSE72094 cohort at 1-, 3-, and 5-year intervals.

UniCox analysis was performed to assess the survival significance of these genes, the identification of 67 genes with a significance threshold set at p < 0.05. Subsequently, a G0A model was constructed based on the 67 survival-related genes (Fig 3A). LASSO and multivariate Cox analyzes were performed on the 13 prognostic genes associated with the G0A cluster. Based on this, we identified six genes (CHCHD4, DUT, LARP1, PTTG1IP, RBM14, and WBP11), and the risk score of the G0A signature was calculated as follows: Risk score = (0.888* exp of CHCHD4) + (0.880* exp of DUT) + (2.067* exp of LARP1) + (1.430* exp of PTTG1IP) + (1.221* exp of RBM14) + (1.589* exp of WBP11). In the training set, the testing set and the TCGA cohort, our Kaplan-Meier analysis revealed a significant improvement in OS among low-risk patients compared to high-risk patients (Fig 3A–3C). We observed that patients classified as high risk generally exhibited lower survival outcomes compared to those classified as low risk in both GSE50081 and GSE72094 (Fig 3D and 3E). The expression patterns of key genes with signatures in the training set, testing set, and TCGA cohort are shown in the heatmap of Fig 3F–3H. The relative expression of six key genes in GSE50081 and GSE72094 is shown in the heat map of Fig 3I and 3J. Additionally, we calculated the area under the curve (AUC) values for 1-year OS, which were 0.718, 0.561, and 0.631, respectively; AUC values for 3-year OS, which were 0.719, 0.569, and 0.683, respectively, and AUC values for 5-year OS, which were 0.718, 0.604, and 0.642, respectively (Fig 3K–3M). Furthermore, we got AUC values for 1-, 3- and 5-year OS duration at 0.633, 0.595, and 0.607 in GSE50081 (Fig 3N) and 0.584, 0.585 and 0.492 in GSE72094 (Fig 3O).

### A nomogram to predict the prognosis of patients

**Fig 4:**
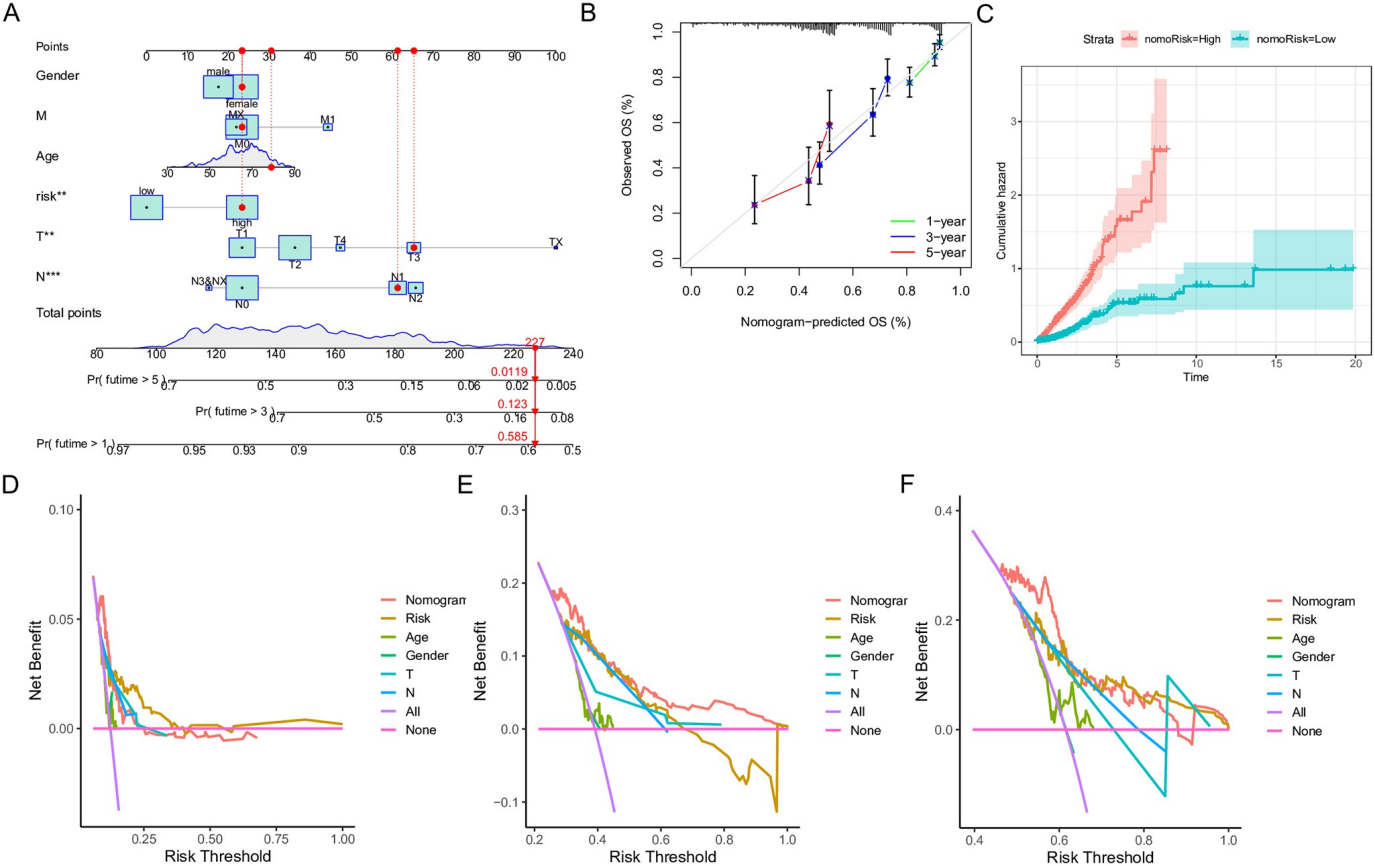
Construction of prognostic nomogram. (A) A nomogram established to predict the prognostic of LUAD patients. (B) Calibration plots included to illustrate the probability of overall survival at 1, 3, and 5 years. (C) The cumulative hazard index of the model. (D-F) Decision curve analysis (DCA) showed the precision of the nomogram in predicting OS 1-, 3- and 5-years among the predictors used.

We developed a nomogram incorporating clinical parameters to address the strong correlation between patient prognosis and risk scores. Using this nomogram, we were able to estimate the OS rates at 1, 3, and 5 years for patients with LUAD (Fig 4A). The calibration curves of the nomogram demonstrated a high level of accuracy between the predicted values and the actual observations (Fig 4B). Furthermore, we evaluated the performance of clinical factors in predicting OS by calculating the AUC values for 1-, 3-, and 5-year (Fig 4B). AUC values were as expected, indicating that the nomogram had excellent predictive ability for prognosis. Furthermore, our analysis showed that the inclusion of various clinical factors in the prognostic model produced greater net benefits in predicting the prognosis (Fig 4C). Furthermore, the DCA results showed that the precision of the nomogram model was the best applied among the factors in predicting 1-, 3- and 5-year OS (Fig 4D–4F).

### Evaluation of TME and immunotherapy prediction

**Fig 5:**
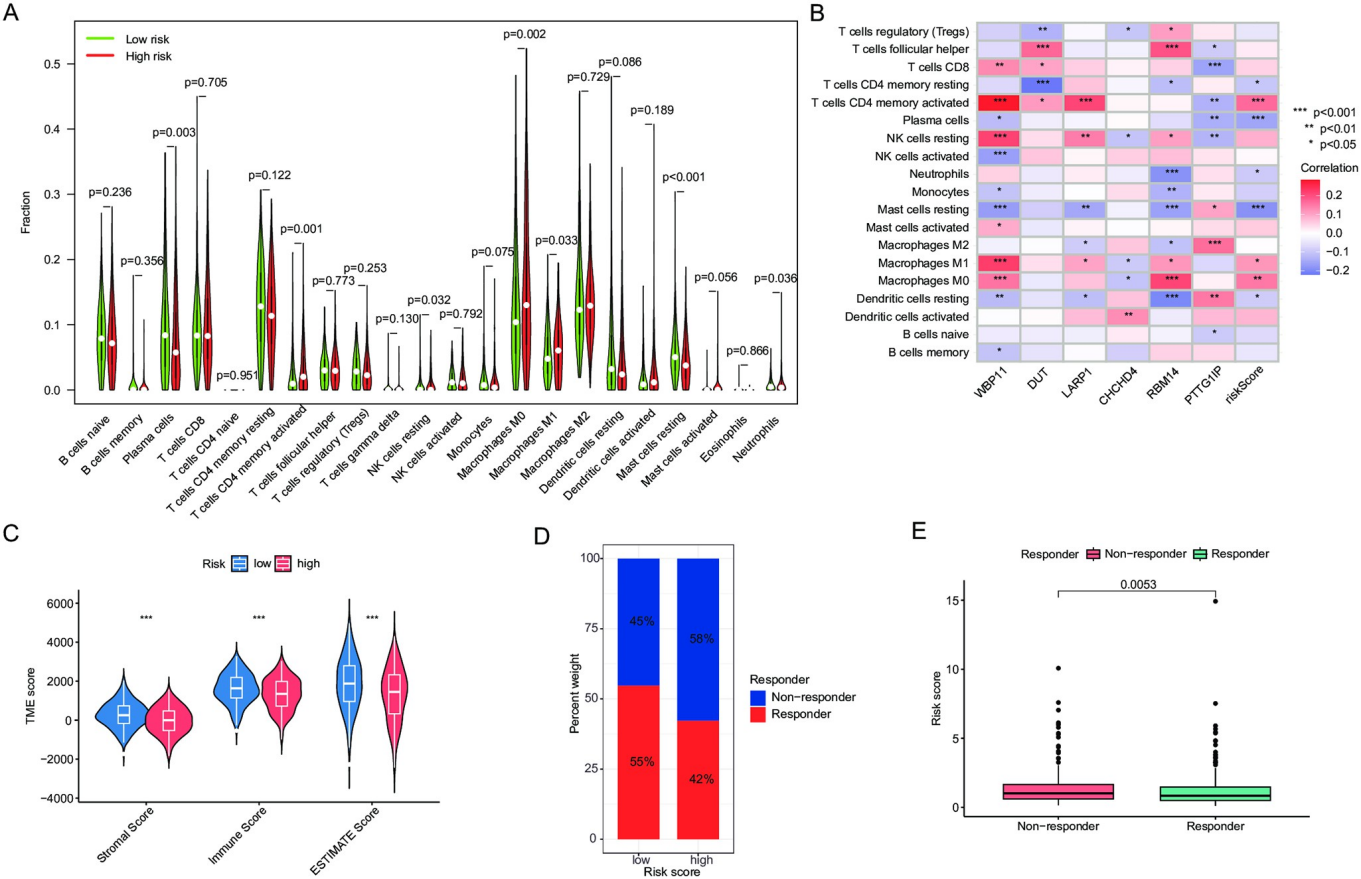
Investigation of immune cell infiltration in the tumor environment. (A) Violin plots to visually depict notable variations in immune cell populations among different subtypes. (B) A correlation analysis to examine the relationship between the risk score and the tumor environment. (C) The stromal, immune, and ESTIMATE scores in different risk groups. (D) Bar graphs of the percentage of immunotherapy response in different risk groups. (E) Bar plots in risk scores between responders and non- responders.

CIBERSORT algorithm analysis showed that activated CD4 memory T cells, neutrophils, M1, and M0 macrophages were up-regulated in the high-risk group, while the low-risk group had higher plasma cells, resting NK cells, resting mast cells, and resting CD4 memory T cells (Fig 5A). Fig 5B illustrates a positive correlation between the G0A model and the infiltration of activated CD4 memory T cells, M1, and M0 macrophages. On the contrary, it shows an inverse relationship between the G0A model and neutrophils, plasma cells, resting CD4 Memory T cells, dendritic cells, and mast cells. Furthermore, we found that the low-risk group exhibited significantly higher stromal scores, immune scores, and estimated scores compared to the high-risk group (Fig 5C). The results of the TIDE analysis showed that patients with high-risk CRS were insensitive to immunotherapy, because there is a low percentage of responders in this group (Fig 5D). Furthermore, these immunotherapy sensitive responders have lower risk scores than non-responders (Fig 5E).

### Target therapy and chemotherapy prediction

**Fig 6:**
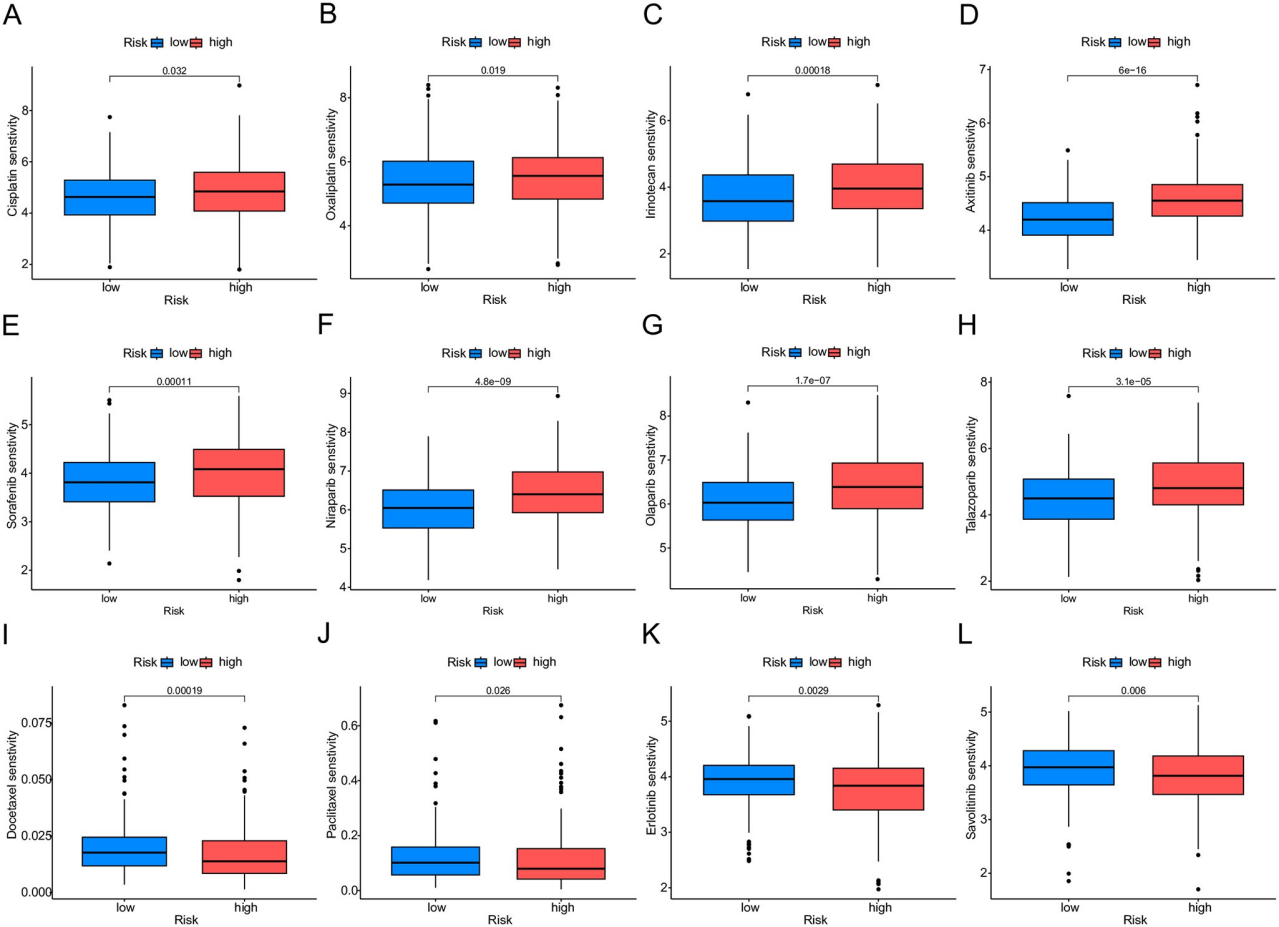
Prediction of drug sensitivity analysis. The drug sensitivity of Cisplatin (A), Oxaliplatin (B), Irinotecan (C), Axitinib (D), Sorafenib (E), Niraparib (F), Olaparib (G), Talazoparib (H), Docetaxel (I), Paclitaxel (J), Erlotinib (K) and Savolitinib (L) in different risk groups.

We evaluated the predictive capacity of the G0A model to determine the response to chemotherapy and targeted therapy in patients with LUAD. Patients with high-risk CRS were resistant to chemotherapy, such as Cisplatin (Fig 6A), Oxaliplatin (Fig 6B), and Irinotecan (Fig 6C). Patients with high-risk CRS were resistant to targeted therapy, such as Axitinib (Fig 6D), Sorafenib (Fig 6E), Niraparib (Fig 6F), Olaparib (Fig 6G) and Talazoparib (Fig 6H). However, this high-risk group was more sensitive to Docetaxel (Fig 6I), Paclitaxel (Fig 6J), Erlotinib (Fig 6K) and Savolitinib (Fig 6L).

### Validation of the G0A signature and the TME heterogeneity in LUAD through single cell RNA sequencing

**Fig 7:**
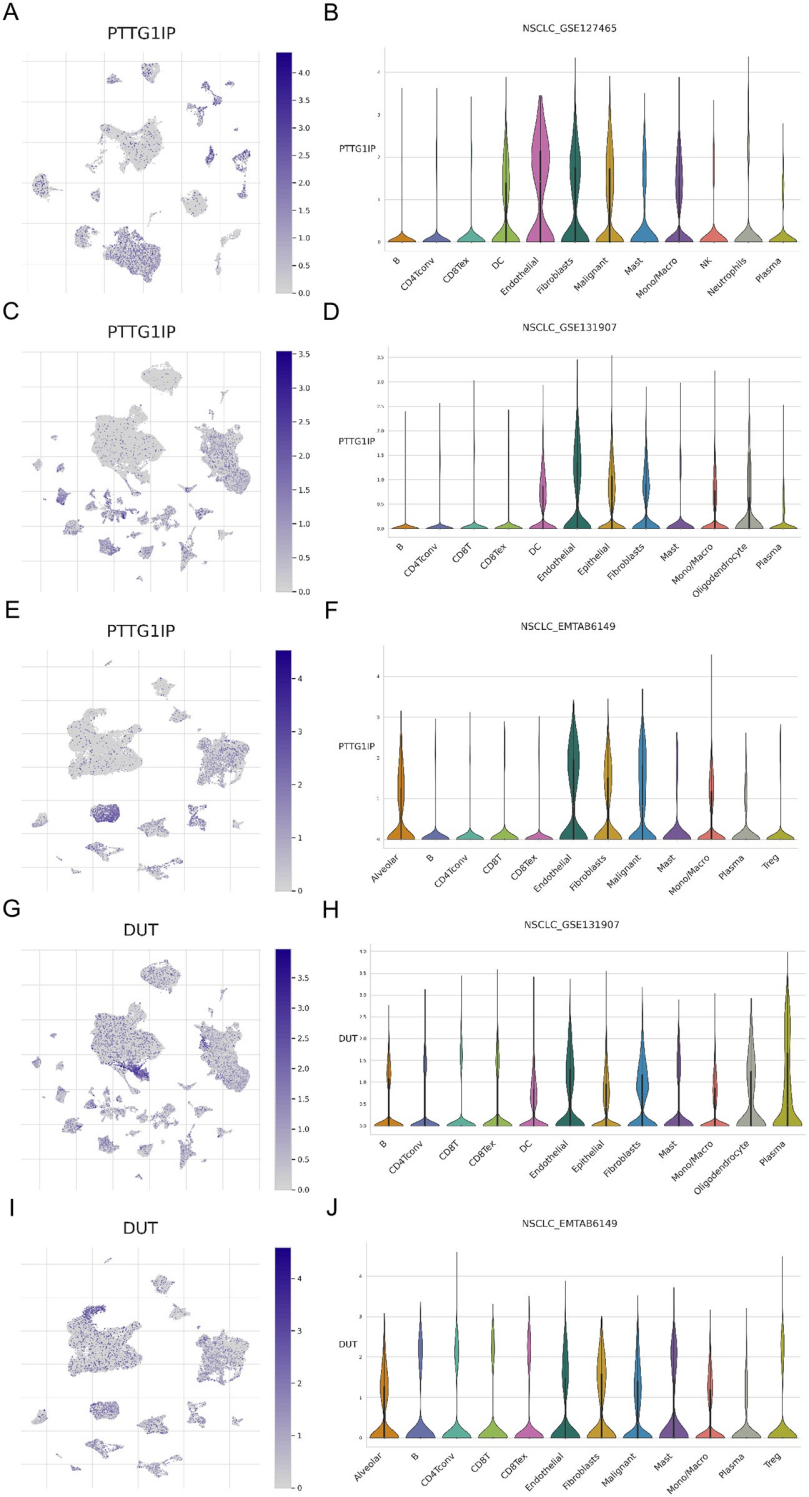
Single-cell transcriptome analysis. (A) and (B), visualization of the T-SNE graph of PTTG1IP in GSE127465, and violin graph of the value of PTTG1IP in different cell types. (C) and (D), visualization of PTTG1IP T-SNE plot in GSE131907 and violin plot of PTTG1IP value in different cell types. (E) and (F), T-SNE plot visualization of PTTG1IP in EMTAB6149, and violin plot of the PTTG1IP value in different cell types. (H) and (I), visualization of the T-SNE plot of DUT in GSE131907 and the violin plot of the PTTG1IP value in different cell types. (J) and (K), visualization of the T-SNE plot of DUT in EMTAB6149 and violin plot of the value of PTTG1IP in different cell types.

To exactly specify which cell types express G0A genes in the tumor microenvironment of LUAD, we used three datasets (NSCLC GSE127465, NSCLC GSE131907 and NSCLC EMTAB6149) of the TISCH database to explore single-cell level analysis using TME. Subsequently, 6 marker genes were analyzed. In all three cohorts, PTTG1IP showed relatively high expression in endothelial cells and fibroblasts Fig 7A–7F. Meanwhile, in the GSE127465 and EMTAB6149 cohorts, PTTG1IP exhibited intriguingly higher expression in malignant cells (Fig 7B and 7F). In both the GSE131907 and EMTAB6149 cohorts, DUT expression was relatively high in endothelial cells and fibroblasts (Fig 7G and 7I). Furthermore, in the GSE131907 cohort, DUT showed relatively high expression in both epithelial cells and plasma cells (Fig 7H). In the EMTAB6149 cohort, DUT exhibited relatively high expression in alveolar cells, malignant cells, and mast cells (Fig 7J).

### The six core G0A-related genes significantly upregulated in cell lines

**Fig 8:**
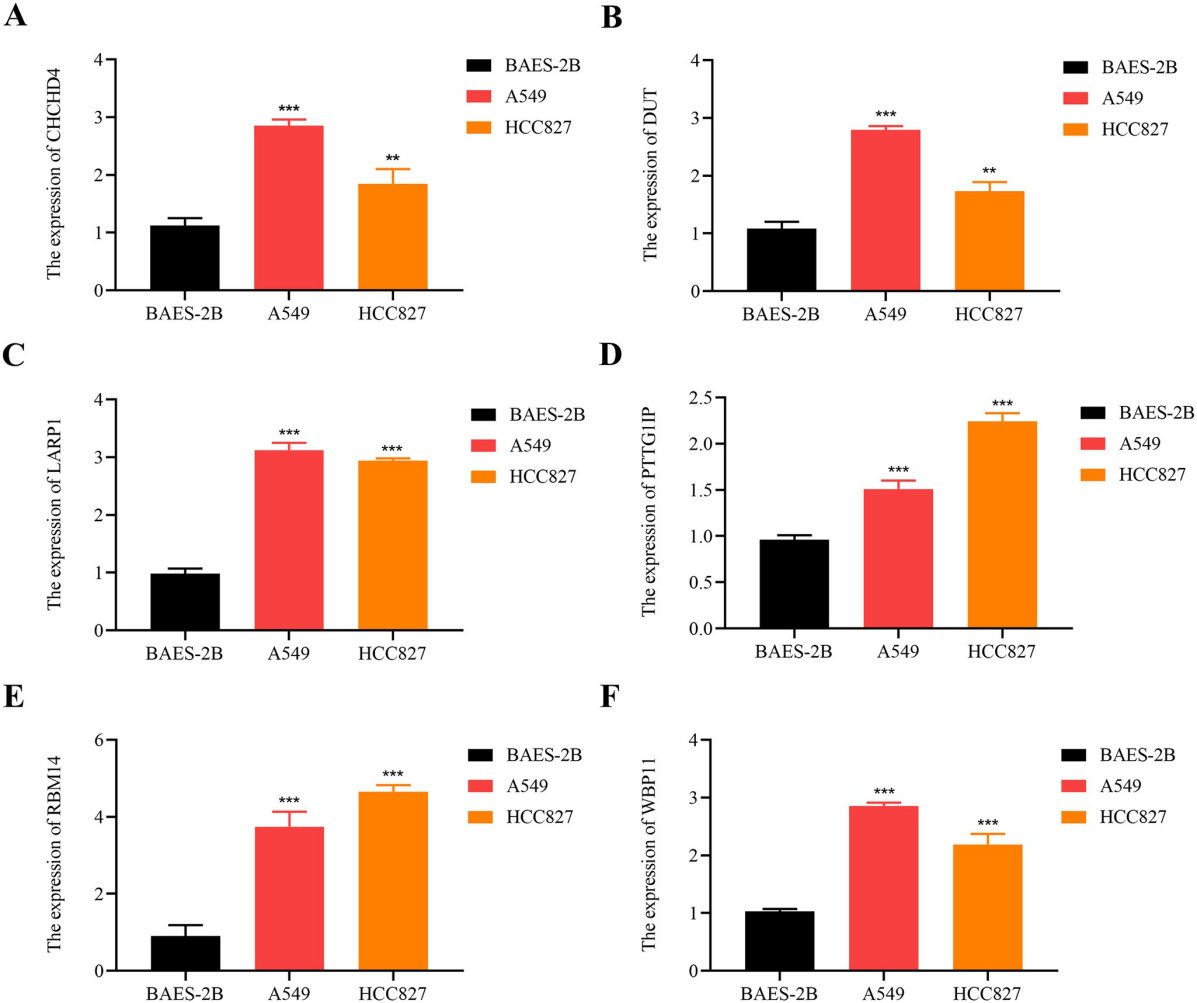
Expression levels of mRNA between the normal lung epithelial cell line BEAS-2B and the LUAD cell lines (A549 and HCC827). (A) CHCHD4, (B) DUT, (C) LARP1, (D) PTTG1IP, (E) RBM14, and (F) WBP11. **, p < 0.01; ***, p < 0.001.

As shown in Fig 8A, compared to BEAS-2B, the expression of CHCHD4 was significantly up-regulated in A549 and HCC827. As shown in Fig 8B, compared to BEAS-2B, DUT expression was significantly up-regulated in A549 and HCC827. As shown in Fig 8C, compared to BEAS-2B, LARP1 expression was significantly up-regulated in A549 and HCC827. As shown in Fig 8D, compared to BEAS-2B, PTTG1IP expression was significantly up-regulated in A549 and HCC827. As shown in Fig 8E, compared to BEAS-2B, the expression of RBM14 was significantly up-regulated in A549 and HCC827. As shown in Fig 8F, compared to BEAS-2B, the expression of WBP11 was significantly up-regulated in A549 and HCC827.

## Discussion

Prognostic models can be used to predict patient survival outcomes, including overall survival and disease-free survival. Prognostic models can assist physicians in identifying high- and low-risk groups of patients, allowing for the application of different treatment intensities based on varying risk levels. Prognostic models can assist physicians in determining which patients are likely to benefit from adjuvant therapy. Prognostic models can assess a patient’s response to specific treatments, particularly immunotherapy. Prognostic modelling can help identify patients who may be resistant to treatment and develop new treatment strategies for these patients. In this study, we established a six-gene signature for LUAD based on G0A-related genes, including CHCHD4, DUT, LARP1, PTTG1IP, RBM14, and WBP11. The CHCHD4, also known as MIA40, gene is a central component of the disulfide relay system. CHCHD4 plays a crucial role in the regulation of the cellular response to low oxygen (hypoxia) and cancer metabolism [31]. Research shows that CHCHD4 drives tumor cell growth, activates the mTORC1 signaling, and influences metabolism mediated by the respiratory chain and complex I biology. It also regulates EMT-related phenotypes in tumor cells [32, 33]. In bortezomib-resistant myeloma cells, there is an increased level of DUT, an important enzyme involved in nucleotide metabolism [34]. Two isoforms of dUTPase have been identified in human cells: DUT-N (nuclear) and DUT-M (mitochondrial) [35]. LARP1 is a conserved RNA-binding protein that interacts with the poly-A-binding protein and regulates the translation of 5’-terminal oligopyrimidine tract (TOP) mRNAs [36]. LARP1 is an oncogene RNA binding protein essential for ribosome biogenesis and cancer cell survival [37]. Upregulation of RBM14 is involved in the regulation of growth, apoptosis, and glycolysis reprogramming in various tumors [38, 39]. WBP11, a pre-mRNA splicing factor, has been identified as a novel protein required for centriole duplication, a process critical for maintaining centrosome number during each cell cycle. Depletion of WBP11 results to centriole duplication defects [40, 41]. The roles of DUT, PTTG1IP, and WBP11 in LUAD are still unclear.

TME is a crucial aspect of tumorigenesis and serves as a therapeutic target for LUAD. The primary components of the TME include stromal cells and immune cells, both of which play a significant role in LUAD progression [42]. Immune and stromal scores have been linked to prognosis and immune microenvironment in LUAD. If the expression levels of the G0A genes in the TME of high-risk patients are low, it may lead to lower stromal and immune scores. ESTIMATE score can also evaluate tumor purity. If the tumor purity of high-risk patients is high, that is, the tumor tissue is mainly composed of tumor cells, and there are fewer normal cells related to the tumor, this may lead to a lower ESTIMATE score [28]. Our study found that higher levels of activated CD4 memory T cells, neutrophils, M1, and M0 macrophages were associated with a high G0A risk score, while higher levels of plasma cells, resting NK cells, resting CD4 memory T cells, and resting mast cells were observed in the low G0A model. This observation suggests that the G0A may influence the composition and function of the TME. Mast cells play a crucial role as a basal stromal component that bridges innate and acquired immunity. Furthermore, they promote the secretion of VEGF, which has been strongly associated with poor prognosis in NSCLC [43]. In our study, we observed that high infiltration of M0 and M1 macrophage cells predicted unfavorable overall survival in patients, particularly in the high-risk group [44]. Furthermore, dendritic cells have shown promising protective effects, and clinical trials have shown that suppression of dendritic cell function in patients with lung cancer. Therefore, dendritic cells can serve as indicators of activated immune responses [45, 46]. To some extent, significantly different expression levels of immunological checkpoints can distinguish low-risk patients, so these biomarkers have been recognized in clinical and research field [47]. These types of immune cells, including mast cells, macrophages, and dendritic cells, have great potential as indicators of immune system activation in the context of LUAD.

Furthermore, the low-risk group showed higher percentages of therapy responders. This suggests that the G0A model can identify patients who are likely to have a more favorable response to immunotherapy. Resistance to chemotherapy is becoming more prevalent in LUAD. In particular, a substantial disparity in drug sensitivity has been observed between the high-risk and low-risk groups. The low-risk group showed a better therapeutic response to chemotherapy drugs, such as Cisplatin, Oxaliplatin, and Irinotecan, but also to targeted therapies, such as Axitinib, Sorafenib, Niraparib, Olaparib, and Talazoparib. However, this high-risk group was more sensitive to Docetaxel, Paclitaxel, Erlotinib, and Savolitinib. Purvalanol A, a Cdc2 / Cdk1 inhibitor, potentiates the cytotoxicity of Paclitaxel on non-small cell lung cancer cells in vitro by targeting Op18/stathmin [48]. Our findings demonstrate that even for patients with high-risk profiles, there are still options available for choosing an optimized therapy. This highlights the importance of personalized treatment in the field of oncology and indicates that it is the future of cancer care.

However, this study has certain limitations. Firstly, we constructed and validated a prognosis signature of G0A-related genes based on the TCGA and GEO databases. Nomograms can serve as clinical decision support tools to help doctors quickly assess the disease risk of patients and communicate the severity of the condition with patients. We will further investigate the clinical significance of the six-gene signature in real-world samples. Secondly, the specific molecular mechanisms by which these core G0A-related genes (DUT, PTTG1IP, and WBP11) mediate the occurrence of LUAD are still unclear and require further research.

## Conclusions

The study conducted by our research team has established a powerful prognostic signature consisting of six genes associated with G0A and specific to LUAD. This gene signature has shown a strong correlation with the prognosis of LUAD and demonstrates the accurate prediction of risk levels. To ensure the reliability and applicability of the signature, we have validated its performance extensively across three independent cohorts, further confirming its prognostic value. Interestingly, our findings suggest that the prognostic ability of this new gene signature may be influenced by interactions among various types of immune cells, including plasma cells, NK cells, CD4+ Memory T cells, mast cells, and macrophages. This observation has important consequences for the diagnosis and treatment of LUAD. However, laboratory intervention in this study is still limited, and more wet experiments are needed to fully understand the relationship between the G0A signature and the tumor microenvironment, and to confirm the validity of our findings.
